# Physician Experience with Direct-To-Consumer Genetic Testing in Kaiser Permanente

**DOI:** 10.3390/jpm9040047

**Published:** 2019-11-01

**Authors:** M. Cabell Jonas, Pim Suwannarat, Andrea Burnett-Hartman, Nikki Carroll, Michelle Turner, Kristen Janes, Christine Truong, Erica Blum-Barnett, Nazneen Aziz, Elizabeth A. McGlynn

**Affiliations:** 1 Mid-Atlantic Permanente Research Institute, Mid-Atlantic Permanente Medical Group, Kaiser Permanente Mid-Atlantic States, Rockville, MD 20852, USA; 2 Mid-Atlantic Permanente Medical Group, Kaiser Permanente Mid-Atlantic States, Rockville, MD 20852, USA; Pim.Suwannarat@kp.org (P.S.); ChristinexTruong@gmail.com (C.T.); 3 Institute for Health Research, Kaiser Permanente Colorado, Aurora, CO 80014, USA; Andrea.N.Burnett-Hartman@kp.org (A.B.-H.); Nikki.M.Carroll@kp.org (N.C.); Erica.Blum-Barnett@kp.org (E.B.-B.); 4 University of Colorado Anschutz Medical Campus, Aurora, CO 80045, USA; Michelle.Turner@cuanschutz.edu; 5 Kaiser Permanente National Quality, Oakland, CA 94611, USA; Kristen.X.Janes@kp.org; 6 Variant Genomics, Inc., Oakland, CA 94611, USA; Nazneen.Aziz@variant-genomics.com; 7 Kaiser Permanente Research, Pasadena, CA 91101, USA; Elizabeth.A.McGlynn@kp.org

**Keywords:** physician survey, genetics, genomics, physician education, direct-to-consumer genetic testing, pharmacogenomic testing

## Abstract

Health systems and physicians nationwide aspire to consistently and reliably apply genetic and genomic information to guide disease prevention, management, and treatment. However, clinical information, including genetics/genomics data from within and outside of the care delivery system, is expanding rapidly. Between November 2017 and April 2018, we surveyed 1502 Permanente Medical Group primary care and specialist physicians to assess the degree to which direct-to-consumer genetic test results were being presented to physicians and identify genetics educational needs among physicians (response rate 15%). Adjusted logistic regression (according to respondent characteristics) was used to calculate adjusted odds ratios (ORs) and 95% confidence intervals (CIs) comparing responses within groups. Results showed 35% and 12% of respondents reported receiving at least one direct-to-consumer health risk genetic result (DTC-health risk) or direct-to-consumer pharmacogenomic test result (DTC-PGx), respectively, from a patient in the past year. Of those receiving at least one test result, 40% (DTC-health risk) and 39% (DTC-PGx) of physicians reported 1+ referral(s); 78% (DTC-health risk) and 42% (DTC-PGx) of referrals were to clinical genetics. In total, 85% of physicians would spend ≥2 h/year on genetics/genomics education.

## 1. Introduction

Clinical knowledge is expanding at a rapid pace, including in the areas of genetics and genomics [1]. Within clinical care, physicians are using genetic testing for diagnosis, treatment, and to guide preventive care. Inside the clinical care delivery infrastructure, health systems and physicians can set the pace for the adoption of genetics/genomics innovations, deciding when and how to implement clinical genetic testing approaches. External to care delivery systems, a robust direct-to-consumer genetic testing market has been established [2,3,4]. Although consumer-directed genetic testing has existed for over a decade, in recent years the cost of these tests has declined to a point of accessibility for most interested consumers, and marketing of these tests has increased consumer awareness [4,5,6,7]. Within the consumer-directed genetic testing industry, companies and consumers are the pace-setters [8]. Direct-to-consumer genetic testing companies such as 23andMe, DNAFit and Color Genomics are providing inherited disease risk information and/or recreational health information to patients [9,10,11]. Companies such as OneOme and Pathway Genomics are offering patients pharmacogenomic results, which aim to inform medication selection and dosing ( in 2018, 23andMe also received approval to offer pharmacogenomic testing) [12,13,14]. These companies are utilizing different business models to provide patients access to genetic information—several companies offer the ability for a consumer to purchase the test without any physician involvement, whereas other companies may require a third-party physician to authorize the test purchase. When physicians are involved in a direct-to-consumer testing purchase, they are typically not part of the patient’s care team or longitudinal care, therefore this type of transaction can be considered direct-to-consumer testing [15,16]. 

Direct-to-consumer genetic testing has raised concerns among physicians due to questions of test accuracy, limited clinical utility, and patient privacy [5,17,18,19,20,21,22,23]. Other concerns stem from utilization of health system and care delivery resources such as educational resources, clinical time with providers (in light of clinical shortages in primary care and genetic counseling), referrals to specialists, and downstream tests and/or procedures to address direct-to-consumer genetic test results shared by patients [17,24,25,26,27,28,29,30,31,32,33,34,35]. Beyond clinical considerations, there is a potential for physician–patient interactions around direct-to-consumer testing to disrupt or erode the physician–patient relationship [36]. Physicians need to strike a balance in embracing patients’ interest in their own care while taking next steps that are appropriate from a clinical perspective.

The direct-to consumer testing industry is still experiencing strong growth [37,38]. Furthermore, a subset of patients is sharing their direct-to-consumer genetic test results with their personal physicians [28,39]. Therefore, health systems must plan for a genetics environment that takes direct-to-consumer genetic testing into account. To aid health systems and physicians in planning around genetics and precision medicine, we conducted a physician survey within all eight Kaiser Permanente regions nationwide. The survey utilized physician reported data to assess how direct-to-consumer genetic test results provided by patients show up within a health system and assessed physician educational needs in handling these and other genetic test results. 

## 2. Materials and Methods

### 2.1. Study Setting

We conducted an electronic survey of providers in eight Kaiser Permanente regions (Colorado, Georgia, Hawaii, Mid-Atlantic States (Maryland, Virginia, Washington, D.C., USA), Northern California, Northwest (Oregon, USA), Southern California, and Washington) from November 2017 to April 2018. Kaiser Permanente is an integrated delivery system that includes more than 12.3 million members nationwide; the overall race/ethnicity distribution of members includes American Indian/Alaskan Native 0.4%, Asian 15%, Black or African American 11%, Hispanic or Latino 24%, Native Hawaiian or Pacific Islander 0.9%, White 46%, Two or More Races 3%. Kaiser Permanente membership demographics in each region reflect the regional populations. The roughly 22,000 physicians within the Permanente Medical Groups provide primary and specialty care within all eight regions, under physician leadership and direction. 

### 2.2. Survey Development and Distribution 

The survey content was informed by review of relevant publications, expert input from primary care and specialist physicians, and input from subject matter experts, the Kaiser Permanente Interregional Genetics Work Group, and the Kaiser Permanente Research Bank leadership team [33,40]. Two rounds of cognitive testing were conducted by the Kaiser Permanente Washington Health Research Institute Survey Research Program. The survey instrument was developed using REDCap Software version 7.6.10, and two rounds of user-acceptability testing were conducted with standard scripts to test survey skip patterns and ensure operability on a variety of electronic devices. 

Physician executive leaders in each region distributed the survey via email to Primary Care (Family Medicine, Internal Medicine), Cardiology/Interventional Cardiology, Gastroenterology, Obstetrics/Gynecology (OB/GYN), Pediatrics, Pediatric Subspecialties, and Medical/Hematology/Gynecologic Oncology. Physicians received a minimum of three email invitations to complete the survey. A 5-point answer scale was used: Strongly Agree, Somewhat Agree, Neither Agree nor Disagree, Somewhat Disagree, Strongly Disagree; numerical scales were also used for relevant questions. Respondents were required to answer physician area of specialty and region; all other questions were optional. To reduce the survey length and time to complete, characteristics of respondents pertinent to clinical practice (physician area of specialty, time in clinical practice, percent adult patients seen, and information on genetics education) were prioritized over personal demographic information, which was not collected [Table 1]. The survey took between 10 and 15 min to complete, and physician responses were anonymous to ensure privacy and encourage full disclosure of information.

### 2.3. Definitions Used

The survey asked about two categories of direct-to-consumer genetic tests: (1) genetic tests sold directly to consumers that scan a person’s genetic makeup for potential health risks (for example, 23andMe, Color Genomics, DNAFit) and (2) pharmacogenomic tests, a subcategory of direct-to-consumer genetic tests that identify genetic variants that may affect drug metabolism, increase risk for an adverse drug reaction, or modify response to a drug (for example, genetic testing for allopurinol (HLA-B*5801) sensitivity before prescribing gout medication). Definitions for both types of tests were provided in the survey. Our survey text instructed users to only consider these types of tests sourcing from outside of Kaiser Permanente when answering questions regarding volume seen in the past year, and to exclude genetic tests ordered as part of clinical care. 

### 2.4. Statistical Analysis

We used univariate and multi-variate logistic regression models to determine the association between current practices in genetics care delivery and providers’ experiences with direct-to-consumer genetic testing with each of the following: physician area of specialty, years in clinical practice, amount of professional time seeing patients, and formal genetics education (Yes/No). Formal genetics education was defined as genetics education in medical school, in other graduate education, in residency or in another relevant setting (genetics/genomics laboratory, research or work experience); no formal/no genetics education included genetics education in undergraduate, CMEs, colleagues, journals, or none. We calculated odds ratios (OR) and 95% confidence intervals (CI) and adjusted for all of the above covariates in addition to Kaiser Permanente region. Years in clinical practice was modeled as a categorical variable (5 years or less, 6–10 years, 11–15 years, and 16 years or more); professional time seeing patients was categorized as: <76% and 76%–100%; received formal genetics education was dichotomized into: Yes, received formal genetics education, or No did not receive formal or any genetics education; percentage of adult patients in the providers’ practice was dichotomized into: 0%–50% or 51%–100%, and physician area of specialty was categorized as: Primary Care (Family Medicine, Internal Medicine), Cardiology/Interventional Cardiology, Gastroenterology, Obstetrics/Gynecology (OB/GYN), Pediatrics, Pediatric Subspecialties, and Medical/Hematology/Gynecologic Oncology. Primary Care (the group with the largest number of respondents) was used as the reference group for between physician area of specialty comparisons. The region with the largest number of survey respondents was used as the reference group for between region comparisons. Survey respondents were permitted to skip questions, resulting in missing data for some items. All analyses were performed using SAS Studio software version 3.7 (SAS Institute Inc., Cary, NC, USA).

### 2.5. Human Studies and Informed Consent 

Study protocols and human subjects’ considerations were reviewed by the Institutional Review Board (IRB) at Kaiser Permanente Mid-Atlantic States; because this survey was primarily aimed at operational quality improvement, it was deemed to be not human subjects research. Nevertheless, when physicians were invited to participate, they were informed that the survey was voluntary and anonymous. The survey included an acknowledgement page where physicians agreed to complete the survey. 

## 3. Results

A total of 9783 Permanente physicians received the survey invitation (43% of total Permanente physicians), 1764 (18%) initiated the survey, and 1546 (16%) completed the survey. Surveys completed by non-physician providers or physician specialists outside of the invited groups were excluded (*n* = 44); 1502 of physicians within the invited groups completed the survey for an overall response rate of 15% [Table 1]. Response rates varied by physician area of specialty with 54% of responses from Primary Care physicians (which combined Internal Medicine and Family Practice), versus 46% of responses from specialists, and by years in clinical practice (17%, 5 years or less; 17% 6–10 years; 20% 11–15 years; 45% 16 years or more). In total, 80% of respondents reported they saw primarily adult patients (>51% of patients seen are age 18 or older). The majority of respondents (86%) reported receiving formal genetics education defined as genetics education in medical school, in other graduate education, in residency or in another relevant setting (genetics/genomics laboratory, research or work experience). 

### 3.1. Direct-To-Consumer—Health Risk Genetic Test Results Received from Patients 

Of those physicians surveyed, 65% reported receiving zero direct-to-consumer health risk genetic test results from patients in the past year. Among those who received these results, most received 5 or fewer, and 5% reported receiving 6 or more results [Table 2]. Among those receiving results, 40% reporting making one or more referrals with the most frequent referral being to clinical genetics (78%) followed by other physicians (22%); more than one referral type could be reported [Table 3]. Gastroenterology, pediatrics and pediatric subspecialties were less likely than primary care to report having received at least one direct-to-consumer health risk genetic test result from patients in the past year; all other specialists were equally likely [Table 4]. Compared to those with formal genetics education, physicians without formal genetics education were less likely to report having heard of direct-to-consumer health risk genetic testing (OR = 0.46; 95% CI: 0.32–0.67) but were equally likely as those with formal genetics education to report having received at least one of these results from patients [data not shown]. 

### 3.2. Direct-To-Consumer—Pharmacogenomic Test Results Received from Patients

Most of the physicians surveyed (87%) reported receiving no direct-to-consumer pharmacogenomics results from patients in the past year. Among those who received these results, most received 5 or fewer results and just 1% reported receiving 6 or more results [Table 2]. Medical/Hematology/Gynecologic Oncology physicians were most likely to receive results and OB/GYN were less likely to receive results compared to Primary Care Physicians [Table 4]. Among those receiving results, 39% reporting making one or more referrals with the most frequent referral being to other providers (58%) followed by clinical genetics (42%); more than one referral type could be reported [Table 3].

### 3.3. Physician Readiness to Make Genetics-Related Referrals 

A majority of physicians responded that they strongly or somewhat agree that they know when to refer patients for genetic consultation (overall = 81%), who to refer to for genetic consultation (overall = 83%), and who to go to with genetic testing questions (overall = 74%; Table 5). Specialists are significantly more likely than Primary Care physicians to strongly or somewhat agree with these questions, with the exception of Cardiology/Interventional Cardiology (when to refer and who to refer to; equally likely as primary care) and Gastroenterology (who to refer to and who to go to with questions; equally likely as primary care). Cardiology/Interventional Cardiology were less likely than Primary Care to know who to go to with genetic testing questions (OR = 0.5; 95% CI: 0.29–0.85). Those in clinical practice less than 5 years were significantly less likely to know who to refer to, and who to go to with questions; those without formal genetics education were less likely to be knowledgeable across all three questions [Table 5]. 

### 3.4. Investing Time in Physician Education on Genetics 

Physician respondents were asked what amount of time they would personally be willing to spend learning how to use the variety and scope of genetic tests pertaining to their practice. Physicians reported being willing to engage in the following hours/year of genetics education: willing to invest 0–1 h/year (15%), 2–4 h/year (51%), 5–7 h/year (16%), and >8 h/year (18%; Table 6). After adjusting for practice length, time spent seeing patients, and genetics education, there was no difference between Primary Care providers and all other providers in the amount of time they would be willing to spend learning how to use the variety and scope of genetic tests pertaining to their practice (*p*-value < 0.05; data not shown).

## 4. Discussion

Our survey sought to establish a set of baseline physician responses regarding direct-to-consumer genetic test results entering our nationwide health care delivery system, in order to guide providers in planning around genetics/genomics and precision medicine. Overall, our results suggest that although primary care and specialty physicians report encountering direct-to-consumer health risk genetic tests and pharmacogenomic test results from patients, the current volume reported by the majority of providers annually is a fairly small proportion of estimated total clinic visits [41,42,43,44]. Our survey found that most Permanente physicians report feeling confident in the processes around identifying and referring patients to genetics for follow up—with specialists generally more confident. Although direct-to-consumer genetic tests are being brought into the health care delivery system by patients at a pace that is manageable, physicians recognize that the areas of genetics/genomics are rapidly growing, and our survey results show that physicians are willing to spend time in obtaining additional genetics/genomics education. 

Although other studies have examined which consumer populations are purchasing direct-to-consumer genetic testing and the consumers’ intentions to share results with their physicians, fewer studies have examined actual sharing behaviors from the receiving physician’s perspective [5,45,46,47,48,49]. The literature reports a range of consumer intentions to share direct-to-consumer results with their physicians; our results support that a subset of patients does share these results with their doctors [36,48]. Consistent with the literature, many patients shared their results with a primary care physician, but we also found that Medical/Hematology/Gynecologic Oncologists were receiving both direct-to-consumer health risk and direct-to-consumer pharmacogenomic tests from patients [50]. In most Kaiser Permanente regions, physicians refer patients to Kaiser Permanente employed clinical geneticists and genetic counselors; regions without employed genetics staff refer patients to partner organizations who provide these services. Physicians follow clinical guidelines to determine when retesting/confirmatory testing is appropriate. The literature reports that, when possible, patients self-refer to genetics to discuss direct-to-consumer results [28]. Within Kaiser Permanente, patients do not self-refer to genetics, a physician makes the referral. This pattern was reflected in the survey results showing that most referrals for direct-to-consumer testing were to clinical genetics. For pharmacogenomic testing, clinical pharmacy and behavioral health referrals were also common, consistent with the target genes tested in pharmacogenomic panels (which typically include genes that influence drug metabolism and inform psychiatric prescribing); this finding solidifies the need to include these departments in planning around processes to manage and triage direct-to-consumer pharmacogenomic test results [13,51,52,53]. Beyond physicians, other clinical personnel supporting patients (including pharmacy technicians, advanced practice nurses, physicians’ assistants, social workers and others) should be considered as groups that would benefit from further clinical education in genetics and genomics. 

Results from published literature consistently indicate that primary care physicians are seeking more genetics/genomics education [17,29,33]. This survey reinforces that physicians continue to view obtaining genetics/genomics education as a valuable investment of time. Our survey offers practical guidance in this area. Among physician respondents, 85% report a willingness to spend two or more hours per year in continuing medical education about genetics. No differences between primary care and specialists were seen in the amount of education desired—indicating that even the most prepared groups are interested in additional genetics education. Currently, genetics education is managed by each Kaiser Permanente region individually, although certain continuing medical education sessions and web-based medical resources are available for all physicians to access. 

Our study has several strengths. First, the survey time period coincided with industry changes that led to a dramatic increase in consumer consumption of direct-to-consumer tests in 2017, including changes in FDA guidance and companies ramping up direct-to-consumer genetic test marketing [4]. Therefore, this survey offers up-to-date information which can be utilized to guide future health system planning [4,47]. Second, our survey included a large sample from physicians in many specialty areas. Prior surveys have focused on the perspectives of primary care physicians, geneticists, or individual specialties; our survey assembles information from several groups into one study. Third, this survey covered a large geographic area, integrating results from physicians in eight Kaiser Permanente regions, which cover eight states and the District of Columbia. 

Despite the strengths cited above, results should be interpreted with the following limitations in mind. Although Kaiser Permanente has a national presence, not every region of the United States is represented. Second, we prioritized specific physician groups to survey and not every physician invited to the survey completed it. Although the study team made efforts to maximize response rates by fielding a mobile and tablet compatible survey, sending the survey from a known clinical leader, and sending multiple email reminders, the overall response rate could be improved. While the distribution of respondents reflects the large proportion of Primary Care, OB/GYN, and Pediatrics providers within the medical group, the number of direct-to-consumer results received by physicians could change with increased physician responses. As direct-to-consumer testing continues to become more mainstream, it may be beneficial to include additional physician groups in the surveyed population. Testing volumes and other results were self-reported by physicians; additional studies may focus on identifying direct-to-consumer genetic test information within the electronic medical record and tracking the referral paths of individual genetic test results (which will be accelerated by electronic medical record solutions that store discreet genetic test results in easily searchable fields). 

When considering these survey results as part of health system planning, it is important to note scenarios in which our findings may underestimate the potential demand on genetics staff from direct-to-consumer genetic testing results. Literature reports that patients would be interested in genetic counseling for direct-to-consumer genetic testing results, if it were available to them [24]. In networks where patients can directly access the genetics department, a higher percentage of patients may directly consult genetics providers (including medical geneticists and genetic counselors) to share results, unless the patients encounter typical barriers to genetics service access [54]. Our survey results indicate that our genetics referrals processes (when to refer, who to refer to, and who to go to with genetics questions) are generally well understood by surveyed Permanente Medical Group physicians, although there is room for improvement. If the referral processes in other health systems are less defined, referrals to genetics and other providers may differ from results reported here. Furthermore, the Kaiser Permanente health plan covers retesting/confirmatory testing when appropriate, according to clinical guidelines and regional processes. Insurance coverage may also impact a patient’s decision to access genetics care or pursue retesting/confirmatory testing. Collectively, providers nationwide are operating with a limited supply of genetic counselors, and therefore this resource should be preserved—whether the business model is an integrated payment and care delivery system (such as Kaiser Permanente) or a fee-for-service care delivery and payment model. Offering physicians clear guidance around when and how to access genetic counselors and geneticists may be warranted to maximize capacity and prioritize the direct-to-consumer results needing specialist attention [24,27]. There are also opportunities to proactively educate patients about direct-to-consumer testing using non-clinical resources such as web-based education, in order to help patients become partners in accessing the right care at the right moment [55]. As more consumer genetic testing companies begin using physician intermediaries as part of the ordering and resulting processes, patients may seek explanatory and health information from these physicians. Future studies in this area would also be beneficial. 

These survey results offer a snapshot of direct-to-consumer genetic test sharing activity reported by physicians within an integrated delivery system and indicate what referrals were made upon receiving the direct-to-consumer test results. As more patients access direct-to-consumer genetic testing and share results with personal physicians in the future, follow-up surveys should be conducted [36]. Whereas today, volumes are manageable, it is notable that over 35% of surveyed physicians report having already received at least one direct-to-consumer health risk genetic test result from a patient in the past year. These results may impact total visits, time spent in discussion within a visit, and patient satisfaction with the encounter [36,43,44]. It is important for all health care delivery systems to consider the impact higher volumes of direct-to-consumer genetic testing results may have on staffing-constrained departments such as primary care and clinical genetics. Assessments of physician and patient satisfaction with the processes and procedures to manage direct-to-consumer genetic test results would also contribute valuable information to the field. Direct-to-consumer genetic testing will impact the clinical infrastructure and physician education needs; proactive planning in these areas will support providers in effectively handling these types of tests today and in the future. 

## Figures and Tables

**Table 1:** Characteristics of survey respondents.

	*N*	%
**Physician Area of Specialty** [required]		
Primary Care (Internal Medicine, Family Practice)	807	54%
Cardiology/Interventional Cardiology	64	4%
Gastroenterology	57	4%
OB/GYN	199	13%
Pediatrics	235	16%
Pediatric Subspecialties	71	5%
Medical/Hematology/Gynecologic Oncology	62	4%
Other	7	1%
**Years in Clinical Practice**		
5 years or less	261	17%
6–10 years	258	17%
11–15 years	301	20%
16 years or more	681	45%
**Professional Time Seeing Patients**		
0%–75%	355	24%
76%–100%	1145	76%
**Percent Adult Patients Seen (>18 years old)**		
0%–50%	307	20%
51%–100%	1194	80%
Received Formal Genetics Education *		
Yes	1300	86%
No	202	14%

* defined in Materials and Methods.

**Table 2:** Physician reported direct-to-consumer health risk and pharmacogenomics test results shared by patients last year.

	How Many Patients Shared Direct-to-Consumer Health Risk Results with You in the Past Year? (*N* = 1467)		How Many Patients Shared Direct-to-Consumer Pharmacogenomic Test Results Completed Outside of Kaiser Permanente with You in the Past Year? (*N* = 1468)	
	***N***	**%**	***N***	**%**
**No patients shared results with me**	958	65%	1281	87%
**5 or fewer patients shared results with me**	433	30%	168	11%
**6–15 patients shared results with me**	73	5%	16	1%
**16 + patients shared results with me**	3	0.2%	3	0.2%

**Table 3:** Self-reported referrals physicians made after receiving direct-to-consumer genetic test results from patients last year.

	Referrals Made to Specialists in the Past Year, for Direct-to-Consumer Health Risk Test Results (*N* = 509)		Referrals Made to Specialists in the Past Year, for Direct-to-Consumer Pharmacogenomic Test Results (*N* = 187)	
	***N***	**%**	***N***	**%**
**Total receiving at least one result**	509	100%	187	100%
I did not refer any patients to a specialist	303	60%	115	61%
Made at least one referral	206	40%	72	39%
**Total referrals made among those who made at least one referral (respondents could refer to >1 provider)**	296	100%	101	100%
Referrals to Clinical Genetics or Genetic Counselors	232	78%	42	42%
Referrals to other providers	64	22%	59	58% *

* Includes Behavioral Health (14%) and Clinical Pharmacy (14%).

**Table 4:** Likelihood that one or more patients shared direct-to-consumer genetic test results in the past year, by physician area of specialty.

	Patients Who Shared Direct-to-Consumer Health Risk Test Results in the Past Year				Patients Who Shared Direct-to-Consumer Pharmacogenomic Test Results in the Past Year			
	**N**	**Zero Patients Shared**	**% 1 or More Patients**	**OR (95% CI)**	**N**	**Zero Patients Shared**	**% 1 or More Patients**	**OR (95% CI)**
**Overall**	1462		35%		1460		13%	
**Physician Area of Specialty**								
Family Medicine/Internal Medicine	782	59%	41%	Ref	781	85%	15%	Ref
Cardiology/Interventional Cardiology	64	72%	28%	0.56 (0.32, 1.00)	64	86%	14%	0.93 (0.45, 1.95)
Gastroenterology	56	75%	25%	0.48 (0.25, 0.90)	57	89%	11%	0.71 (0.30, 1.72)
OB/GYN	196	66%	34%	0.74 (0.53, 1.04)	196	98%	2%	0.11 (0.04, 0.31)
Pediatrics	234	82%	18%	0.33 (0.23, 0.48)	233	90%	10%	0.62 (0.38, 1.00)
Pediatrics Subspecialties	70	77%	23%	0.46 (0.25, 0.82)	71	93%	7%	0.45 (0.17, 1.15)
Medical/Hematology/Gynecologic Oncology	60	48%	52%	1.54 (0.90, 2.64)	58	67%	33%	3.22 (1.78, 5.83)

**Table 5:** Physician comfort with genetic testing processes.

		I Know When to Refer Patients for Genetic Consultation		I know Who to Refer to for Genetic Consultation		I Know Who to Go to with Genetic Testing Questions	
	**N**	**% Strongly or Somewhat Agree**	**OR (95% CI)**	**% Strongly or Somewhat Agree**	**OR (95% CI)**	**% Strongly or Somewhat Agree**	**OR (95% CI)**
**Overall**	1468	81%		83%		74%	
**Primary Role**							
Family Medicine/Internal Medicine	785	72%	Ref	75%	Ref	66%	Ref
Cardiology/Interventional Cardiology	64	75%	1.12 (0.62–2.05)	67%	0.64 (0.37–1.13)	52%	0.50 (0.29–0.85)
Gastroenterology	57	86%	2.46 (1.13–5.35)	86%	2.03 (0.93–4.42)	72%	1.37 (0.72–2.61)
OB/GYN	197	95%	6.91 (3.57–13.37)	94%	4.53 (2.45–8.36)	84%	2.50 (1.62–3.87)
Pediatrics	234	93%	5.29 (3.09–9.06)	95%	6.09 (3.30–11.23)	85%	3.90 (2.48–6.11)
Pediatric Subspecialties	71	96%	8.63 (2.66–27.93)	97%	10.31 (2.48–42.78)	93%	5.41 (2.13–13.76)
Medical/Hematology/Gynecologic Oncology	60	98%	22.55 (3.09–164.60)	97%	8.50 (2.04–35.33)	95%	9.40 (2.78–31.82)
**Years in Clinical Practice**							
16 years or more	664	82%	Ref	85%	Ref	76%	Ref
5 years or less	253	78%	0.80 (0.54–1.18)	76%	0.56 (0.38–0.82)	65%	0.53 (0.37–0.75)
6–10 years	253	84%	1.03 (0.68–1.56)	84%	0.84 (0.55–1.28)	75%	0.87 (0.59–1.27)
11–15 years	298	81%	0.96 (0.66–1.39)	84%	0.90 (0.60–1.33)	74%	0.94 (0.66–1.34)
**Received Formal Genetics Education**							
Yes	1270	83%	Ref	84%	Ref	75%	Ref
No	198	69%	0.53 (0.37–0.76)	74%	0.61 (0.42–0.88)	65%	0.66 (0.46–0.95)

**Table 6:** Hours per year surveyed physicians are willing to spend on genetics education.

	*N*	%
0–1 h per year	215	15%
2–4 h per year	730	51%
5–7 h per year	235	16%
>8 h	255	18%
